# Virtual Obstacle Avoidance Strategy: Navigating through a Complex Environment While Interacting with Virtual and Physical Elements

**DOI:** 10.3390/s24196212

**Published:** 2024-09-25

**Authors:** Fabiana Machado, Matheus Loureiro, Marcio Bezerra, Carla Zimerer, Ricardo Mello, Anselmo Frizera

**Affiliations:** 1 Graduate Program in Informatics, Federal University of Espírito Santo, Vitória 29075-910, ES, Brazil; fabiana.machado@ufes.br; 2 Graduate Program in Electrical Engineering, Federal University of Espírito Santo, Vitória 29075-910, ES, Brazil; matheus.loureiro@edu.ufes.br (M.L.); marcio.bezerra@edu.ufes.br (M.B.); carla.zimerer@ufes.br (C.Z.); ricardo.c.mello@ufes.br (R.M.)

**Keywords:** mixed reality, navigation, smart walker, proxemics, shared control, feedback

## Abstract

Robotic walking devices can be used for intensive exercises to enhance gait rehabilitation therapies. Mixed Reality (MR) techniques may improve engagement through immersive and interactive environments. This article introduces an MR-based multimodal human–robot interaction strategy designed to enable shared control with a Smart Walker. The MR system integrates virtual and physical sensors to (i) enhance safe navigation and (ii) facilitate intuitive mobility training in personalized virtual scenarios by using an interface with three elements: an arrow to indicate where to go, laser lines to indicate nearby obstacles, and an ellipse to show the activation zone. The multimodal interaction is context-based; the presence of nearby individuals and obstacles modulates the robot’s behavior during navigation to simplify collision avoidance while allowing for proper social navigation. An experiment was conducted to evaluate the proposed strategy and the self-explanatory nature of the interface. The volunteers were divided into four groups, with each navigating under different conditions. Three evaluation methods were employed: task performance, self-assessment, and observational measurement. Analysis revealed that participants enjoyed the MR system and understood most of the interface elements without prior explanation. Regarding the interface, volunteers who did not receive any introductory explanation about the interface elements were mostly able to guess their purpose. Volunteers that interacted with the interface in the first session provided more correct answers. In future research, virtual elements will be integrated with the physical environment to enhance user safety during navigation, and the control strategy will be improved to consider both physical and virtual obstacles.

## 1. Introduction

Mobility is the ability to move independently or with the aid of assistive devices, and is directly associated to Quality of Life (QoL) [1,2]. Several health conditions can affect human mobility either permanently or transiently [2], impacting individuals’ daily life. Diseases and pathologies such as stroke [3], multiple sclerosis [4], Parkinson’s disease [5,6], and neurological disorders [4], as well as spinal cord injuries and trauma from accidents, can cause issues such as balance disturbances, weakness, and gait difficulties [7,8].

Moreover, walking difficulties are not limited to impairments caused by diseases and injuries, and may also be associated with aging. As people grow older, they experience physical decline that diminishes their muscle strength, resulting in reduced mobility and difficulties with walking [9]. In addition, there is also a decrease in cognitive and sensory functions, which increases the dependence of elderly people in performing day-to-day tasks [10]. With the global aging population expected to reach 2.1 billion people aged 60 or over by 2050, addressing mobility challenges is becoming increasingly critical [11].

An aging global population is a medical and social demographic issue [12]. Given this trend, there is a need not only for mobility solutions that support independent living [9] but also for practices that promote healthy aging and physical rehabilitation programs [7,13]. Therefore, it becomes necessary to find ways to help, rehabilitate, or compensate the mobility of these individuals. This is extremely important to improve their QoL, independence, and health [14].

Individuals with mobility impairments can utilize augmentative and alternative devices to mitigate the impact of their limitations on daily activities and overall QoL. Mobility aids such as canes, walkers, orthoses, prostheses, scooters, and wheelchairs offer varying levels of support based on the user’s remaining locomotion capabilities [14,15,16].

Among these mobility aids, walkers are designed to enhance natural locomotion by providing stability and support for users with limited motor capabilities [7,14]. They offer a wide base of support, improving balance and stability for those who cannot fully bear weight on their lower limbs [7,15,17]. However, their size can make it difficult to maneuver through doors, narrow environments, and around obstacles. As it is necessary to push/lift it to continue locomotion, they may limit arm movement, contributing to an unnatural gait [14,15].

To address the limitations of traditional walkers, Smart Walkers (SWs) have been developed by integrating robotic and electronic components [14,16]. These advanced features improve user functionality by enhancing gait and cognitive assistance, providing safer navigation, and incorporating fall prevention systems [18,19,20,21]. Additionally, SWs collect valuable data for gait improvement analysis, including health metrics, limb movements, forces, torques, and positional information [7,14,16,22].

Navigating through complex environments can be particularly challenging for individuals with mobility and cognitive impairments [23]. Narrow spaces such as corridors and doorways as well as pathways with obstacles can present significant risks if there is no effective navigation strategy implemented. Without a system designed to avoid collisions and guide the user, these environments can become dangerous and difficult for safe locomotion [24].

Shared control offers a solution to this problem by providing a collaborative approach to navigating such spaces. By integrating advanced guidance systems into assistance devices, shared control facilitates individuals in receiving accurate directions and obstacle avoidance, enhancing both their safety and independence in challenging environments. Shared control and obstacle avoidance is a widely discussed topic in the area of navigation assistance technologies, including SWs. The literature contains many works in which, different sensors have been used to aid in guiding users through potentially hazardous pathways [25,26,27].

However, adding new sensors, controls, and security strategies may cause a cognitive overload when navigating the walker, causing fatigue and discomfort and resulting in increased cognitive demand on the part of the user [28]. The complexity of robotic systems is growing as technology advances and as information exchange among the robot, user, and environment expands; with this added complexity comes the requirement for users to be able to transparently understand the functioning of the robotic system and how to collaborate with it [29].

Integrating various feedback modes can enrich interactions by providing additional layers of engagement. Multimodal feedback systems can be particularly advantageous to users, as not all users adapt in the same way to the same feedback. Various types of feedback can engage multiple senses, offering users increased flexibility and accessibility and making these devices more effective by providing different layouts [30,31].

Another tool that offers more immersive feedback is Mixed Reality (MR). MR lies between two extremities: the real environment, and the virtual environment [32]. In MR, real and virtual objects are presented together in the environment within a single display. One of the major benefits of MR systems is their ability to provide a tool to create training and rehabilitation environments with a lower risk of injury. These systems allow for the manipulation of variables and constraints within complex scenarios, enabling the creation of specific and repeatable conditions that can be analyzed in detail by professionals [33,34].

MR has the potential to enhance user experiences through multimodal feedback as well as to incorporate elements that are not possible in the physical world alone. One such advantage is creating intuitive and transparent interfaces for devices such as walkers [35]. This technology offers a robust solution for enhancing positioning, navigation, comprehension, and monitoring of biomechanical parameters.

The main contribution of this article is to provide enhanced human–robot–environment interaction by demonstrating how control strategies function through comprehensive visual feedback. This approach facilitates user learning by offering clear visual explanations rather than relying solely on verbal instructions, thereby mitigating initial difficulties. Additionally, integrating visual feedback with gamification and serious games transforms the learning experience into a more engaging and less burdensome process, encouraging motivation and reducing pressure [36,37]. Furthermore, the use of MR enables the creation of adaptable virtual environments where obstacles can be dynamically modified to improve the user’s learning experience [38,39]. Unlike real-world scenarios, where physical adjustments are frequently cumbersome, the virtual environment allows for seamless and automatic updates, ensuring a fluid and effective training process with the SW.

To fulfill this main contribution, this article introduces two main aspects. First, the introduction of a virtual obstacle avoidance strategy using a virtual LiDAR sensor allows users to move freely when no collision risks with obstacles are detected. Consequently, users can navigate an empty room with the SW, while in the virtual environment the room is filled with virtual obstacles. The second aspect involves the integration of virtual elements to enhance the user experience through comprehensive visual feedback. This augmentation allows users to observe the operation of the virtual LiDAR and the functionality of the control strategy. Users receive visual cues such as arrows that guide them towards obstacle-free spaces when navigating narrow corridors, entering rooms, and interacting with dynamic or static obstacles.

The combination of these aspects provides immersive feedback that not only clarifies the system’s behavior but also facilitates a more intuitive understanding of the navigation process, thereby improving user confidence and effectiveness in managing complex environments.

## 2. Related Work

Shared control is a strategy that integrates user inputs with automated functions to dynamically adjust the balance of control between human operators and robotic systems. This approach leverages both human decision-making and machine capabilities to enhance navigation safety and user comfort, and is commonly employed in assisted navigation systems to improve human–robot interaction [24,40].

According to Udupa et al. [24], there are several levels of shared autonomy in mobile assistive devices. At one end of the spectrum is manual control, where the user has complete control over the robotic platform without any assistance. The other end, fully autonomous assured safe driving, does not allow the user to control the device; instead, the robot makes all decisions concerning navigation while prioritizing safety and user comfort.

In between these extremes are various levels of shared autonomy control. Robot navigation assistance involves the robot providing support with navigation while the user retains most of the control. Learning-based robotic assistance refers to a system where the robot adapts based on learned user behavior to assist with navigation. Human-aware autonomous tight space maneuvering is a widely used level for indoor navigation. At this level, the user maintains control of the device but receives assistance to maneuver through environments with people and both static and dynamic obstacles. Lastly, in shared control with dynamic user control, the user can make decisions along with the robot and dynamically change their decisions to ensure feasible paths [24].

The control strategy used to assist in safe navigation depends on the user’s residual physical and cognitive capacity. The user may have full control of the walker and be alerted in the event of an obstacle, subsequently making decisions regarding how to deviate from it. In other cases the walker may have full control and safely guide the user through the environment. There may be further situations in which the user has full control until encountering a situation that they cannot deal with, in which case the walker needs to trade or share control in order to avoid an obstacle [28]. Obstacle avoidance strategies are commonly employed in these scenarios to preserve users’ decision-making while ensuring safe navigation, particularly for indoor Smart Walkers.

In the work of Mori et al. [41], the authors used a stereo camera to acquire depth images to calculate obstacles’ distance and position from the walker. With this information, the walker can move to the right or left, and depending on the situation can perform an emergency stop. In [20], the system first establishes a path for the user to follow as a reference. By adjusting the damping parameters of the admittance control, the user feels that approaching the reference path becomes easier, while deviating from it becomes progressively harder. This effect simulates a virtual tunnel of low impedance, making navigation smoother and more intuitive.

In addition to shared control, it is also possible to provide the user with different types of feedback, allowing them to understand how the control strategy works and train in navigating with the robotic platform.

In [42], the authors used two different approaches to locate obstacles and guide the user along an obstacle-free path. In the first approach, eight ultrasonic sensors were used with a vibratory feedback. In the second, TensorFlow was used in conjunction with a webcam, while audio feedback provided obstacle characteristics and suggested a direction to the user. Smart Walkers can also use other sensors to identify and locate obstacles and alert the user, e.g., Light Detection and Ranging (LiDAR) [25,43,44].

Visual feedback can also be integrated into the SW navigation. Ferrari et al. [27] integrated a tablet and a graphical user interface (GUI) into a robotic walker. In this system, the GUI provides the user with visual indications in the form of arrows for navigation support. This feedback can also be used to train the user to move with the walker while respecting and understanding important safety rules. In [45], an ultrasonic sensor was used to measure the distance between the user and the walker, with a Light-Emitting Diode (LED) used to signal when the user was not within the appropriate range.

In recent years, our research group has focused on integrating MR with SWs. Our goal is to enhance the system by providing multimodal feedback and increasing both immersion and interaction. This integration allows us to develop engaging tools such as serious games and to create complex virtual environments without requiring the real environment to be equally complex. This approach offers opportunities for training and ensures safe navigation in a more enjoyable and interactive manner.

Loureiro et al. [35] developed an interface to train Smart Walker users in an MR system. The interface display shows the user the discharged force in the force sensors, the distance between the legs and the walker, and the trunk inclination. By changing the colors of these elements, the display shows the user when these parameters are not ideal. The authors carried out experiments with and without the interface to measure the influence of the visual feedback.

In [46], a multimodal feedback system was designed to assist visually impaired individuals using MR. The system allowed users to explore their environment with the UFES vWalker from an egocentric perspective, providing sensory feedback and physical support while eliminating the risk of real collisions that could cause injury. To validate the system, three shared control strategies with varying levels of assistance were employed. The feedback system comprised two main components: (i) audio feedback delivering auditory cues to guide the user throughout the experiment, and (ii) vibration feedback to alert the user to virtual obstacles.

This article aims to develop a shared control system integrated with Mixed Reality (MR) that allows users to make their own decisions while navigating with a Smart Walker (SW). When an obstacle is encountered, the robotic platform assists with navigation by providing haptic feedback to help the user avoid it. Additionally, the system features a self-explanatory interface with visual feedback and visual cues, enabling users to understand the obstacle avoidance strategy and feel more comfortable learning to use the Smart Walker.

## 3. Methodology

### 3.1. UFES vWalker

**Figure 1:**
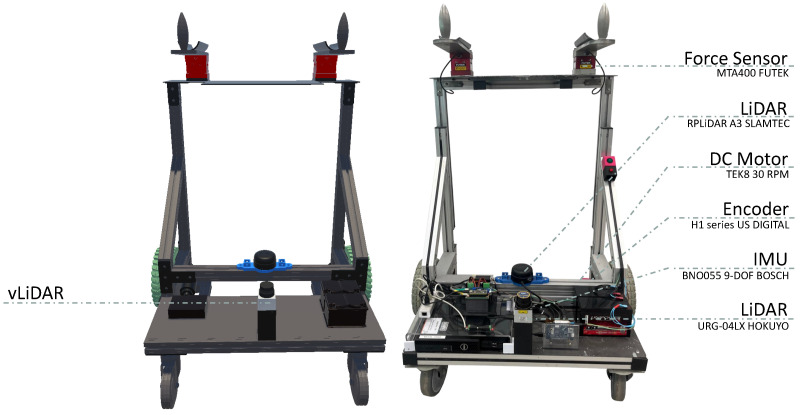
UFES vWalker (**right**) and its digital twin (**left**) with their virtual and physical sensors.

The developed MR system synchronizes virtual and physical environments by connecting the UFES vWalker to a digital twin (Figure 1). The UFES vWalker is a Smart Walker equipped with two DC motors, two encoders (H1 series—US Digital, Vancouver, WA, USA), and an Inertial Measurement Unit (IMU) (BNO055 9-DOF BOSCH, Gerlingen, Germany). These components are based on the user’s movement intentions; they move the walker and acquire position and rotation information, which are then transmitted to the digital twin. This allows the twin to move in a similar manner and provide an immersive experience to the user. The user’s intention is acquired through two force sensors (MTA400 FUTEK, Irvine, CA, USA) located in the forearm supports.

To enhance safety, a Light Detection and Ranging (LiDAR) sensor (RPLiDAR A3 SLAMTEC, Shanghai, China) is aimed at the user’s legs. This allows for estimation of the distance between the user and the UFES vWalker, enabling the walker to move only when the user is in a safe area and bearing its weight at the force sensors to prevent potential falls. The physical environment information is sent to the virtual environment using a LiDAR sensor (URG-04LX Hokuyo, Osaka, Japan) pointing in the direction opposite the other LiDAR. In this way, the user knows whether there are any objects that may pose collision risks even while immersed in the virtual world.

Finally, a virtual LiDAR (vLiDAR) sends information to the UFES vWalker. In this way, navigation strategies that avoid physical obstacles can also be applied to virtual obstacles without the risk of collision. Robot Operating System (ROS) is used for communication between the sensors and PCs. Unity software (version 2020.3.29f1) is used to render the virtual environment, and the ROS# plugin is used to exchange information between environments.

### 3.2. Virtual Obstacle Avoidance Strategy

A strategy to aid in the navigation of narrow environments and to avoid obstacles was developed based on the work of Jiménez et al. [25] and Leica et al. [47]. It consists of identifying virtual obstacles, defined as obstacles generated within the virtual environment (non-existent in the physical world) with a vLiDAR sensor and promoting modulation of an admittance controller to assist in avoiding obstacles, thereby ensuring safe navigation.

To develop this strategy, the idea of proxemic zones is used. The theory of proxemics, created and explored by Hall [48], delves into the examination of human spatial behavior within cultural contexts. The author outlines how people change their interactions in relation to the distance between them. His most notable contribution is the definition of four proxemic zones: intimate, personal, social, and public.

Moreover, concepts of social navigation are integrated into the system, allowing the device to adjust its dynamic behavior depending on whether it encounters a person or an inanimate object. It is essential for the device to anticipate individuals’ reactions, respect their personal space, and adhere to social cues and norms. This ensures that the robot navigates its environment in a manner that is both respectful and responsive to human presence and social interactions [49].

Therefore, two interactions zones were created and characterized as two ellipses with different sizes. The first ellipse is used to calculate the deviation of static obstacles (e.g., virtual walls and virtual cylinders), while the second is used for virtual avatars emulating interactions with other human subjects. Additionally, the elliptical boundaries were intentionally designed to extend slightly beyond the dimensions of the UFES vWalker, allowing clinicians to walk alongside the device without being identified as obstacles.

**Figure 2:**
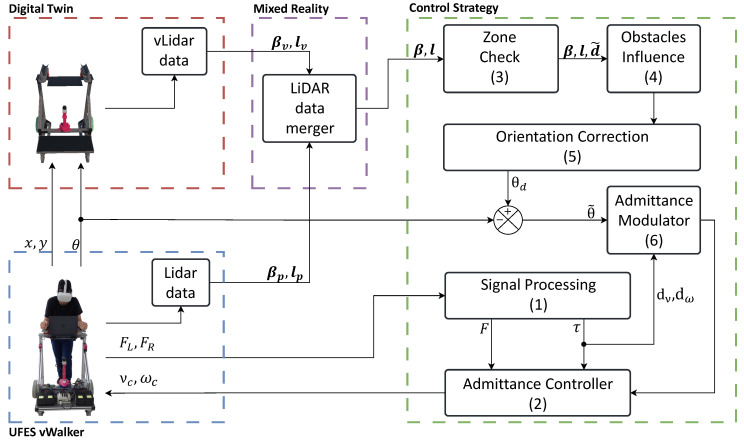
Block diagram containing the proposed MR system together with the obstacle avoidance strategy. The system is divided in four main blocks: the UFES vWalker, digital twin, mixed reality, and control strategy.

The obstacle avoidance strategy was developed using virtual obstacles and integrating virtual and physical sensors. Figure 2 presents the block diagram of the control strategy. Blocks 1, 2, and 6 within the Control Strategy Block are responsible for mapping the users’ movement intentions into the UFES vWalker’s displacement by changing the modulation behavior of the device, as presented in Machado et al. [46] and Sierra M et al. [50].

**Figure 3:**
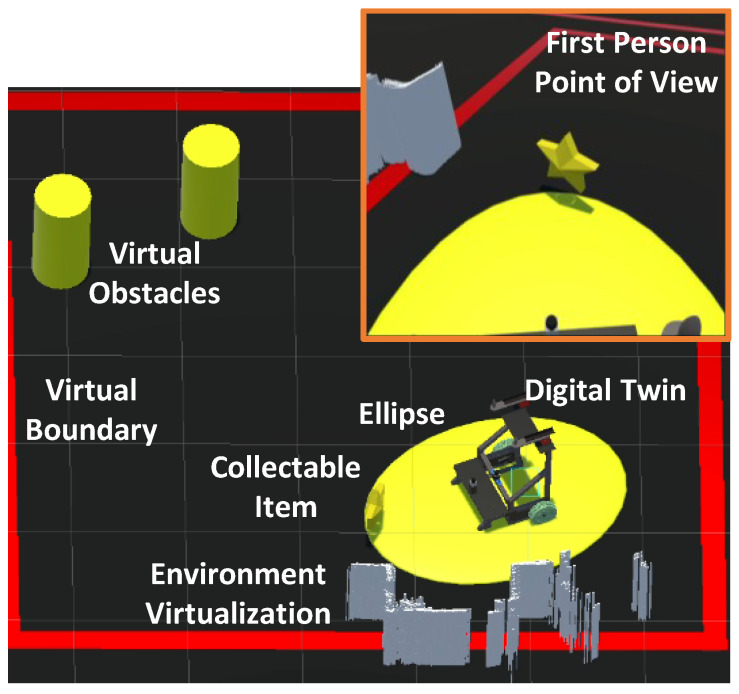
Elements in the proposed MR system, showing the digital twin and the ellipse zone. The white rectangles represent the virtualization of physical elements near the user. The first-person perspective is displayed inside the orange rectangle, highlighting part of the yellow ellipse, a collectible item (a star), and the surrounding white rectangles. The red lines indicate virtual boundaries, which are interpreted as virtual obstacles to prevent the user from crossing them.

The block diagram illustrates the association between the physical and virtual environments. The digital twin receives the values of *x*, *y*, and θ from the UFES vWalker and replicates its movements, providing a sensation of displacement in both the physical and virtual environments. Additionally, MR is enhanced through the interaction between the two LiDAR sensors. Data from the physical LiDAR, including vectors $\beta_{p}$ for laser detections and $l_{p}$ for their respective angles, are transmitted to the virtual environment. These data are rendered such that the physical environment is virtualized and viewed through the HMD, as shown in Figure 3. The laser information appears dense because the points are created and removed less frequently than the sensor readings, resulting in a buffering effect.

**Figure 4:**
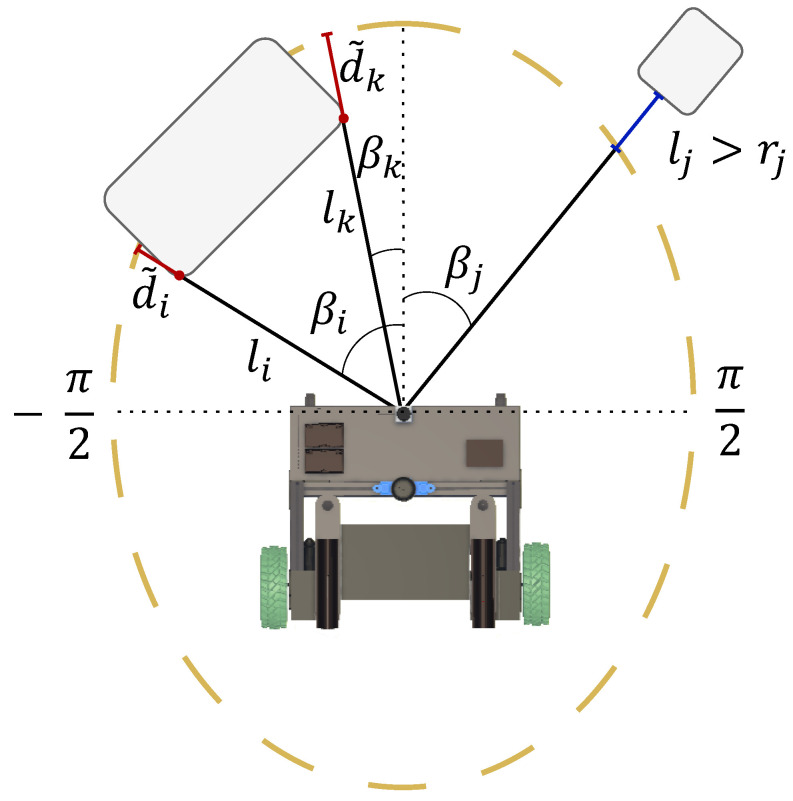
Operation of block 3 (zone check) in the block diagram. For each laser, we verify whether the identification occurs inside or outside the ellipse by comparing the distance r from the center of the ellipse to the edge with the identification distance l for the specific angle β. This check is performed from the limit of β−π/2 to the limit of π/2. The figure shows three identifications: *i* and *k* inside the ellipse and *j* outside the ellipse.

After consolidating the LiDAR data, the output is sent to the first block within the Control Strategy Block to determine whether a collision occurs within the defined zone (ellipse) or not (block 3). Equations (1) and (2) are used to determine whether a collision occurs within the defined zone (ellipse) or not (block 3). Equation (1) calculates the distance *r* from the center of the ellipse to its edge using the angle β. This value is then compared with the distance *l* measured by the sensor using Equation (2). If $\tilde{d}$ is greater than or equal to 0, then the detection occurred within the ellipse, as seen in Figure 4.

(1) $$\frac{cos^{2}\beta }{a^{2}}+\frac{sin^{2}\beta }{b^{2}}=\frac{1}{r^{2}},$$

(2) $$\tilde{d}=r-l.$$

After the information about which rays were detected inside the ellipse has been acquired, it is necessary to calculate the influence of the rays on the deviation strategy (block 4). Obstacles in front of the UFES vWalker have a greater influence than obstacles on its sides. Equation (3) serves to measure this influence, with σ representing the Gaussian width and β indicating the angle of detection. Consequently, the influence of α will be greater for angle values closer to zero, whereas for angles closer to 90° the influence value decreases.

(3) $$\alpha =e^{-{\left(\frac{\beta }{\sigma }\right)}^{2}}$$

To navigate the user safely around obstacles, it is essential to first determine the position of the resulting obstacle. This involves considering the influence of each ray identified within the ellipse. Two weights are assessed: one related to the distance and the other related to the angle.

Equation (4) illustrates the angular influences derived from all detected rays, where *n* represents the number of rays (n=1,2,…,n). This weight, denoted as $W_{\theta }$, is computed by multiplying the Gaussian influence, the angle at which the ray detected the obstacle, and the ratio $\frac{\tilde{d}}{l}$ that indicates the proportion of the collision point that is within the ellipse.

(4) $$W_{\theta }=\sum_{i=1}^{n}\alpha \cdot \beta \cdot \left(\frac{\tilde{d}}{l}\right)$$

Equation (5) is similar to Equation (4) but does not include the β component, highlighting the impact attributed solely to the distance of the ray collision point in relation to the UFES vWalker. Using the ratio between these two weights, shown in Equation (6), it is possible to calculate the angle at which the resulting obstacle is positioned.

(5) $$W_{d}=\sum_{i=1}^{n}\alpha \cdot \left(\frac{\tilde{d}}{l}\right)$$

(6) $$\theta_{o}=\frac{W_{d}}{W_{\theta }}$$

After assessing where the resulting obstacle is, it is possible to guide the user to an obstacle-free path using Algorithm 1. If the obstacle angle value is less than zero, that is, to the left of the walker, then π/2 is added to this value to ensure that the walker deviates to the right. If the value is greater than zero, then the value of π/2 is subtracted t ensure that the walker deviates to the left. In the final case, with the resulting obstacle in the center of the walker, an arbitrary value of π/2 was chosen for the walker to deviate to the left.
**Algorithm 1** Orientation Correction (Block 5)1:**if** $\theta_{o}<0$ **then**2:    $\theta_{d}\leftarrow \theta_{o}+\pi /2$3:**else if** $\theta_{o}>0$ **then**4:    $\theta_{d}\leftarrow \theta_{o}-\pi /2$5:**else**6:    $\theta_{d}\leftarrow \pi /2$7:**end if**

Here, $\theta_{d}$ leaves block 5; after subtracting from the UFES vWalker angle θ (Equation (7)), $\tilde{\theta }$ enters block 6 to modulate the behavior of the UFES vWalker, respectively providing a feeling of ease or difficulty in following that path. It is important to highlight that the logic remains the same regardless of whether an avatar is identified by the SW; the only change is in the size of the ellipse used for calculations. Thus, the controller’s modulation changes depending on the identification.

(7) $$\tilde{\theta }=\theta_{d}-\theta .$$

**Figure 5:**
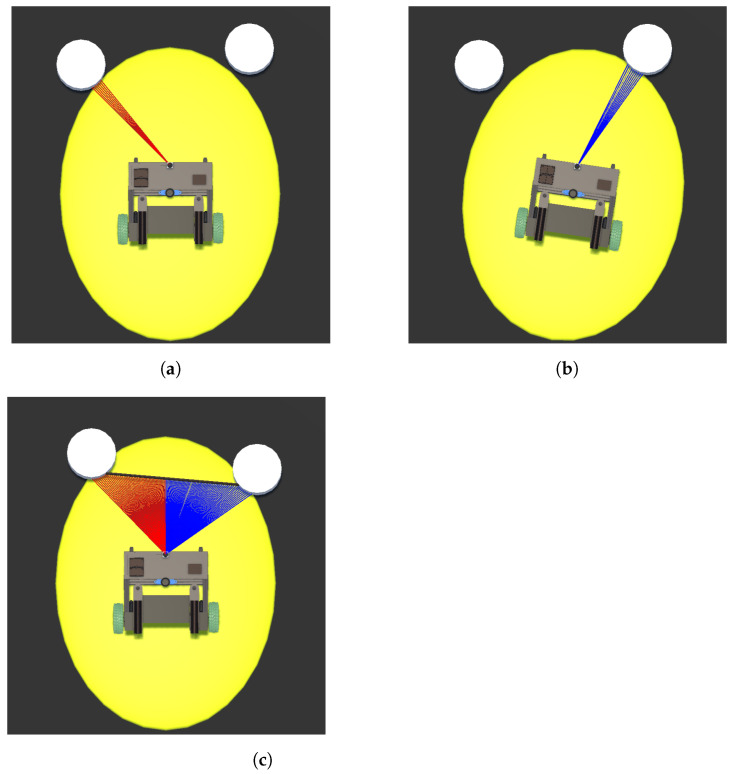
Situation where the UFES vWalker can get stuck between two obstacles. When the obstacle is identified in (**a**), the UFES vWalker facilitates movement to the right. However, when identifying the obstacle to the right in (**b**), the UFES vWalker facilitates movement to the left and the situation in (**a**) is repeated. To ensure safety, the UFES vWalker checks the space between obstacles. In situations that if it is not possible to pass through safely, then the system instantiates a virtual wall, as seen in (**c**).

Due to the logic of the presented algorithm, when there are two symmetrical obstacles with the same number of identifications within the ellipse, the value of $\theta_{obstacle}$ is zero; hence, $\theta_{desired}$ becomes π/2, allowing for the possibility of the walker becoming stuck between obstacles. Another situation where the device can be stuck is depicted in Figure 5. When the obstacle on the left is detected (as seen in Figure 5a), navigating towards the right becomes easier; however, upon identifying the obstacle on the right (as seen in Figure 5b), the obstacle on the left is no longer detected. When trying to go left, the user again encounters the obstacle on the left, resulting in a persistent issue.

To address this problem, an algorithm was developed to detect situations involving simultaneous identification of obstacles on both the right and left sides of the ellipse. Upon such a detection, the algorithm proceeds to measure the Euclidean distance between the inner points of these obstacles. If it is possible for the walker to pass between the obstacles, $\theta_{desired}$ is the same as θ; consequently, $\tilde{\theta }$ is zero, resulting in no modulation. However, if the space between the obstacles is too narrow for the UFES vWalker to pass through safely, a virtual wall is created between the midpoints of each obstacle, as depicted in Figure 5c. When there is no gap between the obstacles, the algorithm consolidates them into a single obstacle, making it possible to calculate $\theta_{desired}$ using the previously outlined method.

Using this strategy it is possible to identify obstacles, estimate the angle of the resulting obstacle, and calculate the desired orientation in order to modulate the admittance controller, thereby ensuring user safety during the operation of the robotic device. It is important to highlight that this obstacle avoidance strategy specifically utilizes virtual obstacles. Physical obstacles are incorporated to provide visual feedback on the physical world within the virtual environment, enhancing the user’s confidence and safety during the experiment.

### 3.3. User Interface

An interface was developed to provide visual feedback on the main elements for the operation of the obstacle avoidance strategy and facilitating greater comprehension of it. The three interface components presented here are ellipses, laser lines, and arrows.

**Figure 6:**
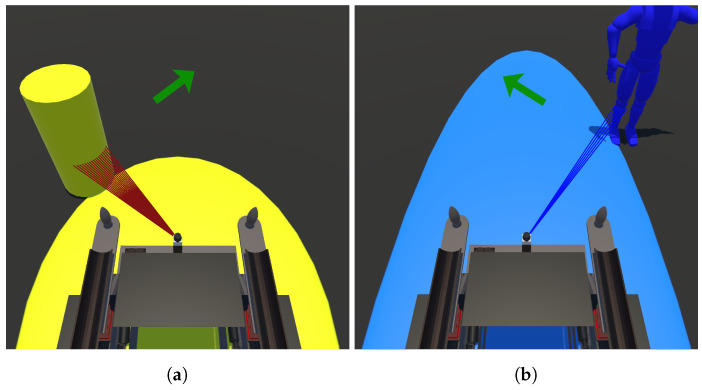
Images depicting the user interface. In (**a**), a static obstacle on the left side of the UFES vWalker is within the yellow ellipse, and the green arrow indicates a suggested path to the right. In (**b**), a person is represented by the blue ellipse, and the arrow advises the user to proceed to the left.

In Figure 6, two ellipses are presented to the user. These ellipses serve to illustrate to the user that when an obstacle interacts within its zone, the UFES vWalker will change its dynamic behavior in order to empower safe navigation. The yellow ellipse shows the area in which static obstacles are considered a potential collision hazard. The blue ellipse indicates the same for an avatar, simulating a walking person, which would happen in a social navigation context where robots must understand and predict human movement patterns in order to interact effectively and safely with people in shared spaces. The two ellipses are shown in Figure 6a,b, respectively. Notably, the yellow ellipse, being smaller, remains visible on the interface at all times, whereas the blue ellipse is conditional upon the detection of dynamic obstacles. Obstacles in the Unity software are distinguished by tags, enabling identification of whether they are static or an avatar.

The laser lines show the user the virtual laser rays that come into contact with the identified obstacles. In Figure 6, it is possible to observe obstacles being identified as well as the rays that collide with them. Finally, the arrow in the top center of the interface in the same figure takes the orientation of $\tilde{\theta }$ to guide the user to an obstacle-free path. When such guidance is unnecessary, the arrow is deactivated.

### 3.4. Experimental Validation

**Figure 7:**
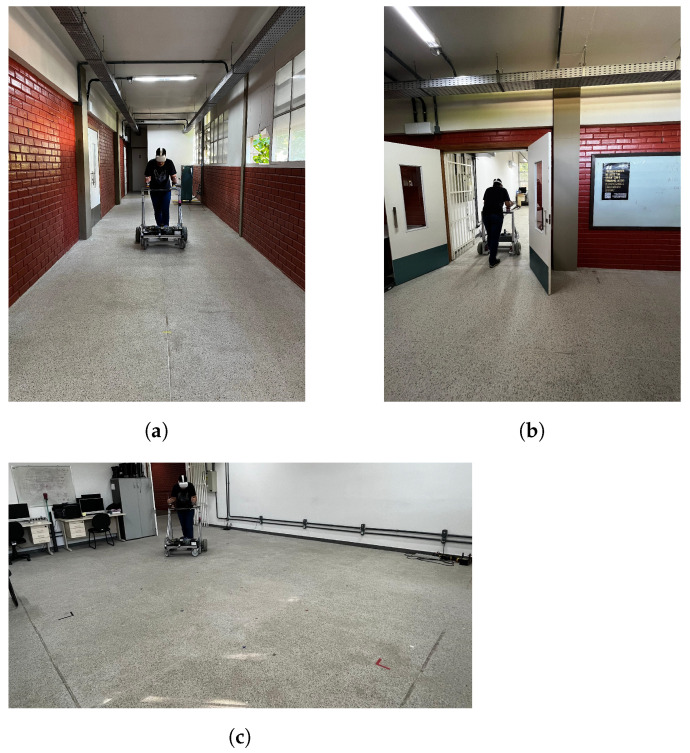
Physical environment in which the experiment took place, consisting of (**a**) a corridor, (**b**) a door with a narrow passage, and (**c**) a room.

An experiment was proposed to test the functionality of the obstacle avoidance strategy and the comprehensibility of the developed interface. The experiment consisted of navigating the UFES vWalker through a corridor, entering a room through a narrow passage (door), and making a circuit inside an empty physical room (Figure 7) with static and dynamic (avatar) virtual obstacles. The volunteers needed to collect all of the stars in order to complete the experimental task.

The experiment was carried out with 20 young and healthy subjects (23.18 ± 2.3 years old; 3 women and 17 men) and consisted of two sessions performed consecutively. In these two sessions, the users had the same objective of collecting stars while navigating the corridor and entering the room. The volunteers were divided into four groups, each of which experienced different conditions for each session (see Table 1). Groups A and B received visual feedback from the interface during the first session; however, only Group B received a brief explanation of the functions of the interface elements (ellipse, laser lines, and arrow). In the second session, the interface was hidden for both groups. Groups C and D began the first session without the interface feedback. In the second session, these groups received feedback from the interface, with Group D also receiving a brief explanation of the functions of the interface elements.

The experiment was structured into an adaptation phase followed by two sessions. As the volunteers had no prior experience with the walker, they were provided with a succinct explanation of how to control the UFES vWalker. Afterwards, they were allowed to navigate using the robotic device until they felt sufficiently comfortable to proceed with the experiment. The only information that was provided to the volunteers was the objective of collecting stars and that they could see physical elements to ensure that they felt safe while navigating with the UFES vWalker. No instructions regarding the obstacle avoidance strategy were provided. At the end of each session, the volunteers answered two questionnaires, which are described in Section 3.6. The experiment was carried out in compliance with the Declaration of Helsinki and received approval from the Research Ethics Committee of the Federal University of Espírito Santo (registration number 6.294.101).

### 3.5. Virtual Environment

The virtual environment used in the experiment was the same as the physical environment depicted in Figure 7. To collect all of the stars, the volunteers were required to walk straight for 10 m along the corridor, make a right turn, and pass through a narrow door measuring 1.63 m wide. When inside the room, which measured 5.1 by 5.7 m, they continued collecting stars while navigating around obstacles and walls until the experiment’s completion.

**Figure 8:**
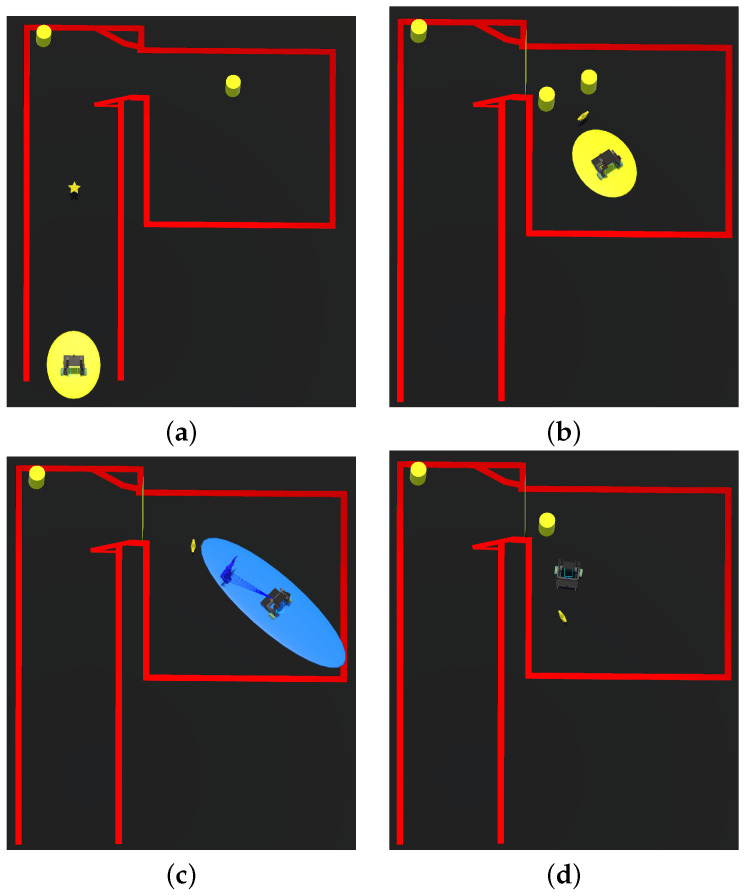
The experiments took place within a virtual environment depicted in (**a**–**d**), showing scenario in which the users performed the tasks. Notably, this virtual environment replicates the physical environment composed of a corridor, a narrow doorway, and a room.

In Figure 8a, it is possible to observe the map from a top view. The figure shows the first star that the user needed to collect, the corridor, two obstacles, the door, and the room where the experiment took place. To integrate the virtual environment, five static obstacles and one dynamic obstacle were created.

The first obstacle was positioned to introduce the volunteer to the obstacle avoidance strategy before entering the room and to guide them towards it. Upon entering the room, another obstacle was placed to the volunteer’s left (Figure 8a), encouraging them to move to the right. The next two obstacles were strategically positioned to activate the virtual wall between them (Figure 8b). The next obstacle, seen in Figure 8c, was a dynamic obstacle; a person’s avatar approached the volunteer in a way that required the use to avoid them. Finally, a last obstacle was located on the user’s right (Figure 8d). After picking up the star related to the last obstacle, the volunteers needed to move around the room again to pick up the last star, after which the task ended.

The obstacles appeared as the volunteers advanced in the task. After collecting a predefined number of stars for each section, the previous obstacle disappeared and the next one appeared. This approach was selected in order to optimize the room’s space while providing a challenging yet navigable environment. Collection of the stars followed the same mechanism; after collecting a star, it disappeared and the next one appeared. This approach facilitated the exploration of different room layouts and obstacle types while enhancing the volunteers’ experience through an engaging and game-like simulation.

### 3.6. System Evaluation

Three evaluation methods were employed in the experiment: task performance, self-assessment, and observational measures. Regarding task performance metrics, the total time to complete the task was recorded along with the duration during which the volunteer engaged the obstacle avoidance strategy. This occurred when the UFES vWalker’s behavioral dynamics were modulated to guide the volunteer in navigating along an obstacle-free path. The self-assessment involved participants completing two questionnaires at the conclusion of each session. Lastly, observational measures were utilized through video recordings to enhance discussions surrounding the MR system and multimodal feedback.

Considering the task performance evaluation, the analysis was divided into intragroup and intergroup comparisons. The intragroup analysis focused on each group’s improvement between sessions. The intergroup analysis compared Group A with Group B and Group C with Group D to assess the impact of the brief explanation of the interface elements on task performance. Additionally, comparisons between Groups A and C and between Groups B and D were conducted to examine the influence of initial exposure to the interface on performance from the first session.

For the self-assessment measure, the first questionnaire was used to evaluate the MR system as a virtual rehabilitation system. The SEQ (Suitability Evaluation Questionnaire for Virtual Rehabilitation Systems) [51] is a questionnaire with 14 questions designed to test VR systems. The questions are used to measure enjoyment, sense of being in the system, feeling of success and control, realism, easy-to-understand instructions, general discomfort, dizziness or nausea symptoms, eye discomfort, disorientation or confusion symptoms, sensor of progress in rehabilitation, perceived difficulty of the task, and difficulty related with the physical interface used in the system.

It is important to emphasize that the experiment did not include individuals who are in actual need of rehabilitation. The primary goal of this study was to technically validate the obstacle avoidance strategy and the navigation system of the UFES vWalker through the integration of physical and virtual sensors. After the system has been validated and deemed safe, experiments with the target population can be conducted safely. Therefore, questions about the rehabilitation process per se required a degree of abstraction from the volunteers when responding. Despite this, retaining the question was essential in order to gather the users’ perspectives, irrespective of the need for the proposed MR system. Along with the responses to other questions, this question is aimed at identifying areas for future improvement.

The second questionnaire was a self-authored assessment tool used to evaluate user interaction with the MR system (UFES vWalker + physical/virtual components) and the developed interface. It comprised two variations: one with two questions about tasks performed when the interface was hidden, and a second with five questions (including the two questions from the first version) designed to evaluate user comprehension of the interface. Both versions are depicted in Table 2.

In the first version of the questionnaire, the first question uses a 5-point Likert scale graded from “I did not feel safe”to “I felt safe”, while the second question is open-ended. The three additional questions in the second version of the questionnaire are all open-ended.

Finally, two cameras were placed at different points in the laboratory, while a third camera that alternated between facing forward (looking at the environment) and backwards (looking at the volunteer’s face) was attached to the UFES vWalker. The video of one experiment is available in the Supplementary Materials.

## 4. Results and Discussion

### 4.1. Task Performance Metrics

Table 3 shows the total time that each group took to complete the session and the time in which the obstacle avoidance strategy was active. The following analysis is conducted both within each group (intragroup) and between groups (intergroup). It is important to note that Groups B and D received a brief explanation of the functions of the interface elements while viewing the interface, whereas Groups A and C did not receive any explanation. The results of each volunteer for each of these groups are shown in Table A1 in Appendix A.

#### 4.1.1. Intragroup Analysis

Group A received visual feedback from the interface during the first session without receiving any explanation of the functions of the interface elements. In the second session, the experiment was conducted without visual feedback. Analyzing Table 3, it can be observed that the average time of execution (TE) for Group A decreased from 431.4 s to 361.06 s from the first to the second session, representing a reduction of 16.3%. Similarly, the time taken to avoid obstacles (TO) also decreased from 80.74 s to 53.41 s, a reduction of 33.85%. This indicates an improvement in both metrics between sessions.

For Group B, which received a brief explanation of the functions of the interface elements during the first session, there was also a reduction in TE from 307.98 s to 267.5 s, for a decrease of 13.14%. In terms of TO, the time was reduced from 59.98 s to 48.4 s, resulting in a reduction of 19.3%.

Group C performed the first session without visual feedback from the interface, then received this feedback in the second session, but without any explanation of the functions of the interface elements. The TE in the first session was 372.58 s, which was slightly reduced by 3.9% to 358.04 s in the second session. A similar pattern was observed in TO, which decreased slightly from 101.27 s to 96.57 s, representing a reduction of 4.64%.

Lastly, Group D also achieved reductions in both metrics, with the reduction in TE being less substantial compared to TO. The TE decreased by 13.43%, from 364.92 s to 315.9 s. In contrast, the TO had a significant improvement, with a reduction of 51.4%, from 102.65 s to 49.89 s.

#### 4.1.2. Intergroup Analysis

An initial intergroup analysis is conducted between groups A and B as well as between Groups C and D, allowing us to examine the impact of the brief explanation of the interface elements on task performance.

Examining the mean and standard deviation of groups A and B, it is evident that group B, which received an explanation about the interface, demonstrates a smaller TE mean and standard deviation in the first session compared to Group A. These two groups managed to maintain a shorter TE in the second session compared to their performance in the first session, even without seeing the interface. The TO values are similar for both groups in the second session, which suggests that Group A understood the interface elements and used this knowledge to improve their performance despite not receiving an explanation. On the other hand, the TE of Group B was lower, indicating that the volunteers spent less time collecting stars in the virtual environment, possibly due to the explanation they received in the first session.

Comparing groups C and D, both groups initially did not receive feedback from the interface and only interacted with the interface during the second session, with Group D also receiving a brief explanation of the interface elements. During the first session, when neither group had access to the interface, the TE and TO were quite similar. In the second session with the interface, Group D experienced a significant reduction in the standard deviation of the TE while Group C saw a less substantial reduction.

The most notable difference was observed in terms of the TO. Group C, which did not receive any information about the interface elements, showed only a slight improvement from one session to the next. In contrast, Group D achieved a reduction of 51.4% in TO. This improvement was particularly evident in the area where the interface offered support, aiding volunteers to avoid obstacles and to navigate along a clear path.

Comparing Groups A and C allows us to analyze the impact of receiving interface feedback during the first session, when there was no explanation of the interface elements. Similarly, comparing Groups B and D provides insights of the same analysis when the interface elements were explained prior to the sessions.

The results shown in Table 3 reveal that group A had a higher mean TE compared to Group C. However, in the second session Group A achieved a greater reduction in both TE and TO than Group C. This improvement in TE and TO for Group A can be attributed to their prior experience with the interface during the first session. On the other hand, Group C may have simply ignored the interface in the second session, as they had already completed one session without it. As a result, Group A’s prior exposure to the interface likely contributed to the more substantial reduction in both TE and TO during their second session.

For Groups B and D, Group B had a lower TE and, notably, a lower TO compared to Group D, which did not see the interface during the first session. In the second session, the TE and TO values for Group D were similar to those of Group B in the first session. This indicates that Group B retained the knowledge acquired from the interface during the first session, leading to further improvements in both TE and TO in the second session. However, the decrease in TE from one session to the next was similar for both groups. Notably, Group D showed a substantial reduction in the standard deviation of the TE, indicating more concentrated data around the TE for all volunteers when the interface was visible.

### 4.2. Self-Assessment Metrics

The SEQ score ranges from a minimum score of 13 to a maximum score of 65. In the first session, the SEQ results were as follows: Group A scored 55, Group B 57.8, Group C 54.2, and Group D 53.4. In the second session, the scores were as follows: Group A scored 57.8, Group B 56.8, Group C 53.2, and Group D 54.8. Analysis of these results indicates that the volunteers found the MR system enjoyable in both sessions, perceived it as realistic, and did not perceive the tasks as difficult. Notably, the results indicate that the volunteers generally expressed satisfaction with the MR rehabilitation system.

Regarding issues detected with MR, none of the volunteers reported symptoms such as dizziness, nausea, eye discomfort, disorientation, or confusion; however, out of the 20 volunteers, six experienced some level of general discomfort at some points during the sessions. Analyzing the responses to the open-ended SEQ question about the causes of their discomfort, it was observed that five volunteers attributed their discomfort to the UFES vWalker, specifically related to forearm support or the sensation of stiffness in using the device, which is associated with admittance controller parameters. Only one volunteer indicated that the discomfort was due to the MR scenario itself.

These results suggest that the MR environment can be a valuable tool when using the UFES vWalker in training or rehabilitation programs. However, it also indicates the need for adjustments to the parameters related to the walker’s ergonomics.

The second questionnaire under assessment consisted of a five-question format designed by the authors, with three questions tailored towards tasks involving the interface. Its objective was to assess the sense of security experienced while navigating with UFES vWalker and to determine the ease of understanding the interface even without explicit explanation of its elements.

The first analysis relates to the three questions that were common to all groups. In the first session, all volunteers from groups A, B, and D expressed feeling secure while navigating the UFES vWalker. In contrast, one participant in Group C answered “somewhat secure” (a rating of 4 on the Likert scale). The subsequent question sought suggestions to enhance perceived security when using UFES vWalker. In Groups A and B, which received visual feedback from the interface in the first session, the responses varied; four individuals proposed no changes, while six offered suggestions such as “gaining more experience with the walker”, “implementing auditory signals to alert of obstacles beyond the field of vision”, and “improving the attachment of the prototype’s arm support”, among others.

In Groups C and D, for the first task seven of the volunteers had no comments, while the other three mentioned “arrows indicating the direction we should go”, “changing color in VR to approach objects”, and “improving odometry”. Notably, two of these suggestions (adding an arrow showing the direction and adding color changes to indicate approaching obstacles) align with the second session, in which the volunteers received feedback from the interface. The last suggestion is valid, as while there are ellipses and laser lines in the interface, there is occasional desynchronization between the physical and virtual environments. Nevertheless, the intention of passing the Hokuyo LiDAR’s sensor information to the virtual environment is to add an additional layer of security and confidence for the volunteers in the task.

For the second session, nine volunteers in Groups A and B felt safe and one voted neutral; the same was the case for Groups B and C. In the next session, the answers remained the same for Group A, while for Groups B and C the suggestions for “improvement in odometry” continued; one volunteer suggested “lighter walker control” and another suggested “more realistic elements of the physical environment within the virtual environment”. All of these suggestions are feasible with the MR system developed, and are extremely important for the future improvement of the system.

The last three questions on the questionnaire were related to the interface seen by the volunteers. The first question was “Can you tell what the purpose of the green arrow shown on the interface is?” In the first session, only Groups A and B interacted with the interface. Among the five volunteers in group B, who received a brief explanation on the interface elements, four identified the arrow as a means to “avoid obstacles” while one associated it with “indicating the direction of the star”. This is not an incorrect answer, as the stars were placed in such a way as to allow the person to avoid the obstacle and collect them; however, a more correct answer would have been in relation to the obstacles. In Group A, comprising five volunteers who did not receive the brief explanation, three mentioned that the arrow indicated the location of the stars, one referenced the “direction to turn”, and the last one noted “avoiding obstacles”.

Consequently, all participants in groups A and B provided satisfactory responses aligning with the element’s intended purpose, even in the absence of an initial explanation. OI the second session, four out of five volunteers in group D accurately described the arrow’s function as either “obstacle avoidance” or “navigation guidance”, while one volunteer stated that they did not know its purpose. In group C, two volunteers mentioned the “direction of the star”, one claimed not to have seen the green arrow, another stated “direction”, which could be the direction in which the UFES vWalker has to turn (this answer was not considered correct), and a final volunteer stated “to turn by itself”, an incorrect response given that the walker requires user movement intention for turning. Therefore, in Group C, which did not receive any explanation about the interface elements, three out of five volunteers could not answer the question correctly.

The second question was “Can you tell what the blue lines shown in the interface were for?” In group B, one volunteer responded that they “indicate the laser ranges”, three volunteers responded that they “show how close the obstacles are”, and one expressed uncertainty. In group A, one volunteer theorized “I thought they were lines from a distance sensor to show an object”, two participants attributed them to “indicating the closeness of obstacles”, and two others reported not observing the blue lines.

In the second session, Group C had four out of five participants respond with “identifying the closeness of an obstacle” or “identification of an obstacle”. One participant mentioned “closeness to the wall”, which is partially correct, as walls are considered obstacles; however, this answer was considered incomplete and was not accepted. In Group D, despite being introduced to the interface, only one volunteer provided a correct response, while two did not perceive the laser lines.

Based on the results, it is clear that the majority of volunteers in Groups A and C associated the blue lines with obstacles, despite not being introduced to the elements. Notably, one participant correctly deduced that these lines function as a sensor indicating the distance to obstacles. In Group B, most volunteers answered correctly. Surprisingly, even with an explanation of the interface, Group D did not provide correct responses to the questions.

The last question was the most challenging, requiring volunteers to discern the purpose of the blue and yellow ellipses. This was difficult because the blue ellipse only became visible when a dynamic obstacle (the virtual avatar) was encountered. Furthermore, volunteers faced the challenge of knowing what an ellipse was and recognizing it from just half of its shape, given that the walker’s design restricted their ability to look backward.

In the first session, all participants in Group B accurately discerned and expressed in their own words that the yellow and blue ellipses served to respectively identify static and dynamic obstacles. In group A, however, no volunteer managed to distinguish between the two. Two volunteers simply stated the purpose as “visualizing obstacles”, two claimed not to have noticed the ellipses, and one volunteer stated that the yellow ellipse represented the robot’s volume while the blue one indicated the intended path when encountering a moving obstacle. This feedback provides valuable insights, such as the recognition that the blue ellipse corresponds to a moving obstacle and, while the yellow one does not represent the robot’s volume per se, it does act as a safety zone taking into consideration the robot’s size.

In the second session, despite having received the introduction, no volunteers from Group D provided a completely assertive response, and their answers were very different. Two said that they did not remember, one mentioned “static obstacles” but did not state anything about the blue ellipse, another said that the ellipse indicated that “there were objects in the way”, and a fourth responded that they “show the area of repulsion around the walker”, which is true, although the yellow and blue elements were missing. Lastly, a final volunteer simply replied “yes” without providing justification. In group C, among the five volunteers one admitted not noticing, while another proposed “safe location radius”, an insightful answer for someone unfamiliar with the interface. Two volunteers said “to limit the walker” and “to indicate the region which I occupied”, which are understandable interpretations considering the yellow ellipse’s consistent presence alongside the walker throughout the experiment. The last participant responded with “virtual obstacles”.

By comparing the responses to the questions about the interface elements to the task performance results, it is possible to differentiate outcomes by group. Analyzing Groups A and B from the first session, it is evident that Group A provided more accurate and confident answers even without an initial introduction to the elements, particularly regarding the ellipses. Looking at Table 3, even without seeing the interface, there is an improvement in TE and TO metrics. This suggests that despite the lack of explanation, the knowledge retained from the first session contributed to better performance in the second session for group A. Group B exhibited a similar trend, although the volunteers in group A provided more correct answers than in group B. This was unexpected, as group B received a brief explanation about the interface; however, for Group A the elements appeared to be self-explanatory.

In contrast, Groups C and D, which received interface feedback only in the second session, showed a different trend. When analyzing the first question about the function of the arrow, most participants in group D answered correctly, while those in Group C did not. This difference may explain the difference in TE and TO values from the first to the second session. Using the arrow, the volunteers could determine which direction to turn, thereby avoiding obstacles. Consequently, Group D managed to reduce both the TE and TO parameters, while for Group C the values remained similar between sessions. Despite receiving an explanation, the majority of volunteers in Group D did not answer questions about the other elements correctly. This could be due to the volunteers not retaining the information throughout the session. This discrepancy might be attributed to their prior experience with the virtual environment. In the first session, these volunteers did not need to interact with the interface to complete the session; thus, in the second session, they may not have deemed the interface to be significant and simply replicated their approach from the first session. Nevertheless, Group C provided good responses regarding the laser lines; therefore, it seems that the arrow was the most crucial element for understanding the obstacle avoidance strategy, which may explain why Group C did not see a significantly improvement in the TE and TO metrics.

### 4.3. Observational Metrics

Throughout the experiments, observations were documented for subsequent analysis and videos were captured from various perspectives to validate the data obtained through the previously described metrics.

One situation that occurred occasionally involved desynchronization between the walker’s odometry and the virtual environment. At times, the environment became more challenging to navigate when this desynchronization was significant; consequently, the volunteers had the added task of not only avoiding virtual obstacles but also exercising caution in seeking to prevent collisions with physical obstacles in the real world. In any case, this error was already minimized using the fusion of the implemented odometry with visual odometry as well as by utilizing mapping and localization algorithms.

Another scenario experienced by volunteers from all groups, particularly those in groups C and D, was surprise at seeing the dynamic obstacle (avatar) approaching the UFES vWalker and sometimes not being able to avoid it. Nevertheless, in such cases the obstacle was automatically deactivated. It was also possible to observe that in the second session the volunteers were already accustomed to the approaching avatar and were able to avoid it more easily. Moreover, it was interesting to note that certain volunteers from subgroups A and C noticed and asked about the function of the interface elements. They were curious to know what the elements meant, and by analyzing the questionnaire results they were able to identify the elements without the need for an explanation.

The proposed interface and obstacle avoidance strategy does not restricted users from making their own decisions; even if the interface suggests going left, users are free to choose to go right instead, albeit with increased difficulty imposed by the controller. During a particular event that involved avoiding a double obstacle, a virtual wall was instantiated between the two obstacles to indicate the impossibility of passing through. While the interface predominantly suggested a leftward direction, the star was positioned to the volunteer’s right. Only one volunteer adhered to the suggestion to go left, turn around, and then collect the star. The majority of the volunteers exerted force to make the UFES vWalker turn to the right until the algorithm identified the resulting obstacle on the left and suggested that the user go to the right, thereby facilitating movement toward the star.

## 5. Conclusions

This article’s main contribution lies in presenting and validating a novel technique for multimodal human–robot–environment interaction that integrates MR with a Smart Walker (SW). The MR system combines physical and virtual sensors to enable shared control with an SW. A key aspect of this strategy is the virtual obstacle avoidance system, which utilizes a virtual LiDAR sensor and adjusts the admittance controller to facilitate safer navigation. This article also explores how integrating an interface with the key components of the proposed strategy enhances understanding of the system by providing comprehensive visual feedback in an immersive scenario.

Our analysis of the results indicates that our volunteers found the MR system enjoyable, experienced little to no confusion, did not perceive the tasks as difficult, and felt secure while performing the experiment. Regarding the interface, volunteers who did not receive any introductory explanation about the interface elements (groups A and C) were mostly able to guess their purpose. Group A, which interacted with the interface in the first session, provided more correct answers. The arrow in the center of the screen and the laser lines were mostly understandable. However, the ellipse caused confusion, and its objective was not transparent to the volunteers.

Comparing the questionnaire responses and task performance metrics across the groups reveals distinct patterns. Groups A and B showed improvement in the TE and TO metrics from the first to the second session, with Group A performing more accurately and confidently even without an initial explanation of the interface elements. This suggests that for Group A the elements were self-explanatory, contributing to better performance. Conversely, Groups C and D, which only received interface feedback in the second session, displayed different results. Group D answered the questions about the arrow’s function correctly and showed reduced TE and TO values, while Group C, in which most volunteers did not know the arrow’s function, did not see similar improvements despite understanding the laser lines element. This indicates that the arrow was an important element for understanding the obstacle avoidance strategy, which likely impacted Group C’s performance.

The experiment revealed how different conditions affected user perception. By using questionnaires and dividing participants into groups, we gathered important information on how to improve the virtual environment and identified factors that facilitated or hindered navigation and enhanced interaction with the UFES vWalker. This feedback will allow us to make necessary adjustments to improve the user experience. In addition, conducting the experiment with individuals without impairments helped to ensure the system’s safety and reliability before testing with the target population, allowing for further adjustments to provide better support.

In future research, virtual elements will be incorporated into the physical world to increase user safety when navigating and to provide volunteers with interaction with more real elements. Furthermore, it is essential to improve the odometry synchronization of all the systems involved. Moreover, the control strategy needs to consider physical obstacles and potential overlaps between physical and virtual elements in order to enhance human–robot–environment interaction.

Finally, tests with the target population need to be conducted n order to encourage continuity of gait rehabilitation as well as to understand the effects of the MR system, both in terms of the affective aspects and possible symptoms of cybersickness. In order to sustain the flow state and keep the user engaged, it is essential to maintain a balanced relationship between challenge and skill, thereby preventing boredom or anxiety [52]. MR systems can help in this process by adding playful elements to the environment and allowing for the construction of more or less complex scenarios, helping to keep rehabilitation motivating and promoting patient improvement.

## Figures and Tables

**Table 1:** The experiment comprised four distinct conditions for each group: first, whether or not they initially viewed the interface; second, for those who viewed the interface, whether or not they received a brief explanation of the interface elements (arrows, laser lines, and ellipses).

Groups	First Session	Second Session
A	Visible Interface No Brief Explanation	Hidden Interface //
B	Visible Interface Brief Explanation	Hidden Interface //
C	Hidden Interface //	Visible Interface No Brief Explanation
D	Hidden Interface //	Visible Interface Brief Explanation

**Table 2:** Self-authored questionnaire. The first two questions were asked regardless of the performed task, while the final three were exclusively asked to users who interacted with the interface during the task.

Question-Second Questionnaire	
Q1.	Did you feel safe using UFES vWalker?
Q2.	What could be done to increase your sense of security?
Q3.	Can you tell what the purpose of the green arrow shown in the interface is?
Q4.	Can you tell what the blue lines shown in the interface were for?
Q5.	Can you tell what the ellipses (yellow/blue) shown in the interface were for?

**Table 3:** The mean and standard deviation for the time of execution (TE) and time spent for obstacle deviation (TO) parameters for groups A, B, C, and D for both sessions.

Groups (Mean ± Std)	First Session Visible Interface		Second Session Hidden Interface	
	TE (s)	TO (s)	TE (s)	TO (s)
**A**	431.4 ± 115.55	80.74 ± 22.19	361.06 ± 105.58	53.41 ± 18.40
**B**	307.98 ± 53.58	59.98 ± 26.27	267.5 ± 59.68	48.40 ± 32.92
	**Hidden Interface**		**Visible Interface**	
**C**	372.58 ± 114.43	101.27 ± 58.87	358.04 ± 82.80	96.57 ± 59.95
**D**	364.92 ± 100.11	102.65 ± 75.42	315.9 ± 35.16	49.89 ± 21.58

## Data Availability

The original contributions presented in the study are included in the article, further inquiries can be directed to the corresponding author.

## Supplementary Materials

The following supporting information can be downloaded at: https://www.mdpi.com/article/10.3390/s24196212/s1, Video S1: Video of an experiment showcasing the UFES vWalker moving in the physical environment while assisting the volunteer in avoiding virtual obstacles, the digital twin navigating in the virtual environment, and the proposed interface being displayed to the volunteer.

## Abbreviations

The following abbreviations are used in this manuscript:

GUI	Graphical User Interface
LED	Light-Emmiting Diode
LiDAR	Light Detection and Ranging
MR	Mixed Reality
QoL	Quality of Life
SW	Smart Walker
vLiDAR	virtual Light Detection and Ranging

## Appendix A

**Table A1:** Time of execution (TE) and time spent for obstacle deviation (TO) for each volunteer in Groups A, B, C, and D for both tasks. The mean and standard deviation values for each group are presented as well.

Group-Volunteer	First Session Visible Interface		Second Session Hidden Interface	
	TE (s)	TO (s)	TE (s)	TO (s)
A-V1	352	72.45	248.3	31.92
A-V2	464	84.44	430	51.13
A-V3	274	51.8	244	41.55
A-V4	548	113.8	426	78.71
A-V5	519	81.78	457	63.76
**A** **(mean ± std)**	**431.4 ± 115.55**	**80.74 ± 22.19**	**361.06 ± 105.58**	**53.41 ± 18.40**
B-V6	306.9	42.74	252.1	19.88
B-V7	325.8	75	236.9	69.28
B-V8	318.4	72.95	261.3	51.48
B-V9	221.3	15.95	217.2	11.5
B-V10	367.5	88.3	370	89.83
**B** **(mean + std)**	**307.98 ± 53.58**	**59.98 ± 26.27**	**267.5 ± 59.68**	**48.40 ± 32.92**
C-V11	542	119.98	483	175.7
C-V12	416	186.54	345	126.26
C-V13	330	49.92	262.1	13.77
C-V14	339.8	107.72	384	79.02
C-V15	235.1	45.22	316.1	88.09
**C** **(mean ± std)**	**372.58 ± 114.43**	**101.27 ± 58.87**	**358.04 ± 82.80**	**96.57 ± 59.95**
D-V16	512.8	230.22	340.6	35.81
D-V17	385	110.92	361.9	24.01
D-V18	380.5	68.05	310	48.71
D-V19	292.3	58.86	290	61.96
D-V20	254	45.23	277	79
**D** **(mean ± std)**	**364.92 ± 100.11**	**102.65 ± 75.42**	**315.9 ± 35.16**	**49.89 ± 21.58**
